# Identifying clinical course patterns in SMS data using cluster analysis

**DOI:** 10.1186/2045-709X-20-20

**Published:** 2012-07-02

**Authors:** Peter Kent, Alice Kongsted

**Affiliations:** 1 Research Department, The Spine Centre of Southern Denmark, Lillibaelt Hospital, Institute of Regional Health Services Research, Member of the Clinical Locomotion Network, University of Southern Denmark, Middelfart, Denmark; 2 Nordic Institute of Chiropractic and Clinical Biomechanics, Member of the Clinical Locomotion Network, Odense, Denmark

**Keywords:** Outcomes assessment, Back pain, Cluster analysis, Text messaging

## Abstract

**Background:**

Recently, there has been interest in using the short message service (SMS or text messaging), to gather frequent information on the clinical course of individual patients. One possible role for identifying clinical course patterns is to assist in exploring clinically important subgroups in the outcomes of research studies. Two previous studies have investigated detailed clinical course patterns in SMS data obtained from people seeking care for low back pain. One used a visual analysis approach and the other performed a cluster analysis of SMS data that had first been transformed by spline analysis. However, cluster analysis of SMS data in its original untransformed form may be simpler and offer other advantages. Therefore, the aim of this study was to determine whether cluster analysis could be used for identifying clinical course patterns distinct from the pattern of the whole group, by including all SMS time points in their original form. It was a ‘proof of concept’ study to explore the potential, clinical relevance, strengths and weakness of such an approach.

**Methods:**

This was a secondary analysis of longitudinal SMS data collected in two randomised controlled trials conducted simultaneously from a single clinical population (n = 322). Fortnightly SMS data collected over a year on ‘days of problematic low back pain’ and on ‘days of sick leave’ were analysed using Two-Step (probabilistic) Cluster Analysis.

**Results:**

Clinical course patterns were identified that were clinically interpretable and different from those of the whole group. Similar patterns were obtained when the number of SMS time points was reduced to monthly. The advantages and disadvantages of this method were contrasted to that of first transforming SMS data by spline analysis.

**Conclusions:**

This study showed that clinical course patterns can be identified by cluster analysis using all SMS time points as cluster variables. This method is simple, intuitive and does not require a high level of statistical skill. However, there are alternative ways of managing SMS data and many different methods of cluster analysis. More research is needed, especially head-to-head studies, to identify which technique is best to use under what circumstances.

## Background

Much clinical research is focused on the outcomes achieved by patients and usually such outcomes are collected at standardised time points over a follow-up period. For example in back pain research, it is routine practice to measure pain and activity limitation at time periods such as 3, 6 and 12 months after the initial contact with the patient.

Recently, there has been interest in using the short message service (SMS) or text messaging, on cell phones to provide a more detailed assessment of a patient’s clinical course [1,2]*.* An example is using weekly SMS messages sent from an automated service to each patient’s cell phone requesting they reply with the number of days of bothersome pain experienced over the previous week. The technology for using this method is becoming common, inexpensive and widely used in some research settings.

Although SMS provides less detailed information than can be obtained by longer validated questionnaires, an advantage of SMS data is that this high frequency of measurement can provide a much more detailed view of an individual’s clinical course. This is particularly useful in the study of conditions such as back pain, which commonly is a fluctuating, recurrent condition [3,4], because the infrequent measurement points that have traditionally been used to measure outcomes may not be representative of a patient’s actual clinical course. To illustrate: it could be that a single measurement, such as a 3-month outcome, occurs during a brief period in which the patient was uncharacteristically pain-free or occurs during a short-lived relapse. Either way, that single measurement would not be representative of the patient’s typical clinical course. In the context of a clinical trial, this may not be overly problematic, as averaging outcomes across a group is likely to smooth out this effect. However, in the context of research questions that focus more on the outcomes of subgroups or individuals, this issue of representativeness may be more problematic.

SMS has shown acceptable reliability. Johansen and Wedderkopp [1] compared patients’ self-reported clinical course information obtained by SMS with the same information obtained via telephone interview. For patient recall of ‘how many days they had problems due to low back pain’ over the previous week, they found an average difference between the two methods of 0.0 days (95%CI −1 to 0.9 days). For the same question with a one-month recall period, the average difference was 0.7 days (95%CI −4 to 5 days).

Having gathered outcomes information using frequently repeated measures such as SMS, it is not clear what methods are optimal for identifying clinical course patterns in these data. Statistical approaches for analysing SMS data are relatively novel. One approach is to calculate the area-under-the-curve (AUC) for individuals and average these data for the group being studied. This will give some summative information of health impact and is particularly suitable when comparing groups, for example in a randomized controlled trial. However, currently there is considerable interest in the clinical and research communities in whether clinically important subgroups of patients with nonspecific LBP can be identified [5] and in that context, such ‘whole group’ statistical approaches to SMS data may not be the most suitable. The aim of subgrouping is to identify homogenous subgroups of people who have particular treatment needs or distinct prognoses [6-8], and there is some evidence that subgrouping LBP can improve patient outcomes and reduce the costs of care [9,10].

There are a number of research methods being explored to detect the presence of such subgroups and determine whether or not they are clinically important. One approach is to observe differences in the clinical course patterns (trajectories) of participants in research studies, including clinical trials or longitudinal cohort studies [8,11-15]. It is not known yet whether these clinical course patterns will actually assist in the identification of clinically important subgroups or instead, whether they are just a novel form of outcome measurement.

Methodological approaches to subgrouping LBP clinical course patterns using longitudinal SMS data measured at high frequency are only starting to be explored and, to our knowledge, only two published articles have described such methods. Both describe novel and interesting approaches. The first article by Kongsted et al. [13] used a visual assessment method where the researchers viewed each individual patient’s clinical course and interpreted it using a clinically intuitive framework of patterns. This method has the advantages of simplicity and high clinical interpretability but has three disadvantages: (i) it requires the formation of very specific decision rules so as to not require subjective judgments when classifying individuals, (ii) it requires an interpretative framework that is necessarily arbitrary, and (iii) it is inefficient when dealing with large datasets. The second article by Axen et al. [11] used cluster analysis, an automated form of pattern recognition, to identify clinical course patterns.

Cluster analysis is a group of statistical techniques designed to find latent patterns of scoring within datasets [16]. These techniques seek to find a class structure (in this case, a set of clinical course patterns) that optimally explain the variability in the way that people have scored in the cohort. In statistical terms, in this context they seek to find the class structure that maximises the variance between all identified clinical course patterns and minimises the variance within each clinical course pattern.

In the study by Axen et al. [11], a traditional form of cluster analysis was used (hierarchical cluster analysis, Ward’s method followed by K-means cluster analysis [17]) to decide the optimum number of clinical course patterns that best fitted their SMS data. This is an important decision as it determines which patterns will be described and the cluster membership of each participating patient. However in hierarchical cluster analysis, this decision is imprecise as it requires a subjective judgment from the researchers using an arbitrary criterion (stopping rule) and different stopping rules can produce different results [16]. In contrast, there are more recent forms of cluster analysis that use probabilistic (Bayesian) methods to determine the optimum number of patterns and therefore do not require this subjective judgment. Examples of such methods are Two-Step Cluster Analysis *(SPSS Statistics/IBM, Chicago IL, USA)* and Probabilistic data mining *(Monash University, Melbourne, Australia*). A similar probabilistic approach is available in Latent Class analyses *(Statistical Innovations, Boston MA, USA),* where decisions about number of patterns can be based on statistical testing. A potential disadvantage of removing a subjective judgement is that some identified clinical course patterns may be mathematically appropriate but not clinically relevant. For example, the prevalence of a pattern may be too low to be clinically useful or the distinctions between two patterns may not be clinically relevant. However, it is possible to join cluster memberships together, if post-hoc analysis reveals this to be appropriate.

**Figure 1:**
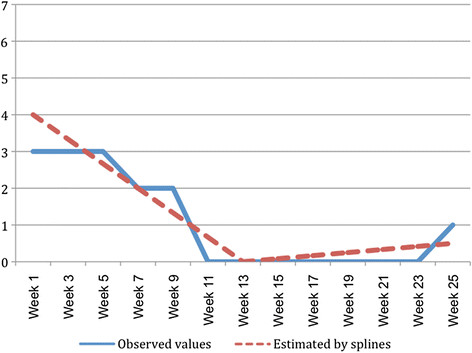
Hypothetical example of an individual’s SMS data (days per week with pain) that has been transformed by spline analysis into regression coefficients.

An additional feature of the method used by Axen et al. [11] was that, instead of clustering the SMS data in its original format (days of bothersome pain measured weekly), they first used spline analysis to create regression coefficients of each individual’s clinical course pattern and then clustered those coefficients. A hypothetical example of an individual’s SMS data that has been transformed by spline analysis into regression coefficients is shown in Figure 1. The authors defended this approach by arguing that it was not practical to use all the 26 weeks of data that they had collected. However, it is not obvious why it would have been impractical to use the SMS data in its original format. Furthermore, although spline analysis may have advantages in some circumstances, it is a ‘dumbing down’ (data reduction) of the available information based on statistical assumptions which may not always be met. For example, the spline analysis approach used by Axen et al. [11] required an assumption that the regression coefficients of two straight lines would adequately describe the clinical course pattern of each individual. Perhaps for some back pain patients in primary care, this assumption is valid, but for patients with fluctuating clinical courses or patients who do not improve, this may not be a valid assumption. As cluster analysis of SMS data in its original form does not require such assumptions, there was a need to investigate whether this was a practical alternative and if it offered other advantages.

Therefore, the aim of this study was to determine whether Two-Step Cluster Analysis can identify clinical course patterns distinct from the pattern of the whole group, by including all SMS time points in their original form. It was a ‘proof of concept’ study to explore the potential, clinical relevance, strengths and weakness of such an approach.

## Method

### Study design

This was a secondary analysis of longitudinal SMS data collected in two randomised controlled trials conducted simultaneously from a single clinical cohort.

### Participants

A dataset was constructed by combining the weekly SMS data collected in two randomised controlled trials. The cohort formed in this way was not intended to be generalisable but was constructed purely to illustrate the potential of a novel statistical approach. These two clinical trials were conducted in a non-surgical secondary-care department within a government-funded spine centre in the Health Region of Southern Denmark. This outpatient department specialises in the multidisciplinary assessment and treatment of spine pain. One trial (ClinicalTrials.gov NCT00454792) compared the efficacy of an exercise program compared with a restriction of high-level physical activity, in a cohort of people with both nonspecific LBP and MRI-identified Modic changes [18]. In the other trial (ClinicalTrials.gov NCT00459433), the efficacy of usual care was compared with usual care plus additional psychosocial intervention in a cohort of people with nonspecific LBP but no MRI-identified Modic changes (unpublished). Recruitment into the two trials occurred concurrently, with the additional inclusion criteria of a minimum LBP intensity of 3/10 on an 11-point numeric rating scale, a pain episode between 2 and 12 months and participants’ age being between 18 and 60 years. Within both trials, the treatment groups were comparable at baseline and there were no significant differences in outcomes between treatment groups at any time period. Therefore, these data (n = 332) were pooled for the purpose of the current study. Apart from descriptive baseline characteristics obtained from patient completed questionnaires, only two variables from those data were included in the current analysis; days of back pain problems over the previous week and days of sick leave due to back pain over the previous week. Both variables were measured on a 0 to 7 day scale.

Written informed consent was obtained from each participant at recruitment into either trial and ethics approval was obtained for both trials from the Regional Ethics Committee. In the ethical framework of our governmental health region, the secondary analysis performed in the current study did not require additional ethics approval.

### SMS questions

Each week, for 52 weeks, participants were sent the following two questions by an automated SMS service *(*http://www.sms-track.com*).* The first question was “Using a number from 0 to 7, please answer how many days in the last week you have had problems due to lower back pain”. Upon receipt of the answer to that question, a second question was sent “Using a number from 0 to 7, please answer how many days you have been off work because of your lower back pain this week. (Answer with X if you are not working)”. The automated SMS service stored responses in an electronic database at the time they were received. Time off work (sick leave) was subsequently recoded so that 5 or more days out of a maximum of 7 all meant a maximum of 5 working days of sick leave.

A reminder text message was automatically sent to any participant who had not answered within five days. A research secretary phoned all participants who had three or more missing answers to determine if there were any technical barriers to participation.

### Data analysis

The statistical clustering technique used was the Two-Step Cluster Analysis *(SPSS Statistics/IBM)* which, in addition to determining the optimal number of natural classes (clinical course patterns) within the data, also classified each participant into one of the identified patterns. An innovative feature of the analysis in the current study is that each SMS data time point was entered as a separate variable into the analysis. However, as cluster analysis is a multivariable statistical technique, consideration needed to be given to the risk of ‘overfitting’ the data [16]. Overfitting is present when an analysis excessively fits the available data and therefore has limited generalisability outside of the available sample. Classification overfitting can occur due to an inadequate sample size relative to the number of variables, due to a lack of representativeness of the participants, and due to the presence of an excessive number of ‘noise’ variables. There is considerable debate about overfitting in statistical classification and a lack of consensus about appropriate sample size ratios for cluster analysis. However in other forms of multivariable analysis, authors have argued for a minimum of 10 events per independent variable to avoid overfitting [19,20]. If this were applied to the current context, this would be an equivalent of 10 participants per variable. Therefore, as the current sample contained 332 participants, we included the SMS data from every second week (fortnightly data) – 26 variables in the cluster analysis. In a sub-analysis to explore the effect of reducing the SMS frequency, these data were re-analysed using monthly data – 13 variables in the cluster analysis.

**Figure 2:**
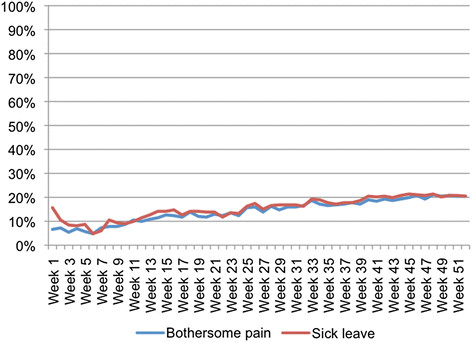
Proportion per week of missing SMS responses.

Participants were asked to answer these SMS messages every week, but as some people did not reply every week, there were 14.2% missing data overall. The proportion of missing data increased over the 12-month period, as illustrated in Figure 2. Like many multi-variable statistical techniques, Two-Step Cluster Analysis does not tolerate missing data and would have excluded any person with any missing data. Such an approach to missing data in this study would have resulted in the sample being reduced to n = 145, a loss of 56.3% of the cohort. Therefore, multiple imputation *(SPSS Statistics/IBM)* was used to impute missing data prior to cluster analysis. All the available 52 weeks of SMS data for pain was used in the imputation of pain data and similarly for sick leave data.

For each clinical course subgroup that was identified, the proportion of people classified into that cluster was calculated. The median (25–75 quartiles) raw scores for the whole group and each clinical course pattern were also calculated for each fortnightly time point. These data were then graphed as visual representations of the clinical course patterns.

As Two-Step Cluster Analysis assumes a normal distribution of interval data, and as the raw SMS data did not meet this criterion, it was Log10 transformed to approximate normality before the cluster analysis occurred. However, the raw SMS data were used when determining the median and interquartile ranges for the clinical course patterns.

Many clustering techniques as diverse as traditional cluster analysis [21], Two-Step Cluster Analysis [17] and latent class analysis [22], p242], have an assumption of independence of the clustered variables. This refers to a low correlation (collinearity) between the variables used to form clusters. This collinearity can take the form of global correlation (between the variables entered into the analysis) and conditional correlation (conditional on membership in one or more clusters). Global correlation can be easily calculated but conditional correlation requires diagnostics specific to the cluster analysis technique used. As high frequency data such as SMS are likely to have some collinearity, Pearson correlation matrices were constructed for the SMS data used in the analysis of both the fortnightly and monthly time intervals. To describe the global collinearity in these SMS data, the mean, standard deviation and range of these correlations were reported.

Multiple imputation, cluster analysis and correlations were performed using SPSS Statistics version 19.0.0 (IBM, Chicago IL, USA). All other analysis was performed using Excel 2008 for Mac version 12.2.8 (Microsoft Corporation, Redmond, WA, USA).

## Results

### Cohort characteristics

The demographic characteristics of the cohort are shown in Table 1.

**Table 1:** Characteristics of cohort participants

**Characteristic**	
Number of participants	332
Age *(mean)*	40 (SD10, range 18–61)
Gender *(proportion women)*	58%
Pain intensity *(Numerical Rating Scale 0–10, mean)*	
Low Back Pain (now)	5.0 (SD2.2)
Low Back Pain (worst last 14 days)	7.7 (SD1.9)
Low Back Pain (average last 14 days)	5.8 (SD1.7)
Leg pain (now)	2.2 (SD2.6)
Leg pain (worst last 14 days)	4.0 (SD3.3)
Leg pain (average last 14 days)	3.1 (SD2.6)
Activity limitation *(Low Back Pain Rating Scale 0–100, mean)*	50 (SD16.6)
Depression *(Beck Depression Inventory, scale 0–63, mean)*	10.1 (SD6.8)
Duration of present LBP episode at baseline	Range 3–12 months
Occupational physical activity *(mainly):*	
Sitting	46 (14%)
Mostly walking	77 (23%)
Walking and some lifting	91 (27%)
Heavy work	118 (36%)

### Clinical course of pain

**Figure 3:**
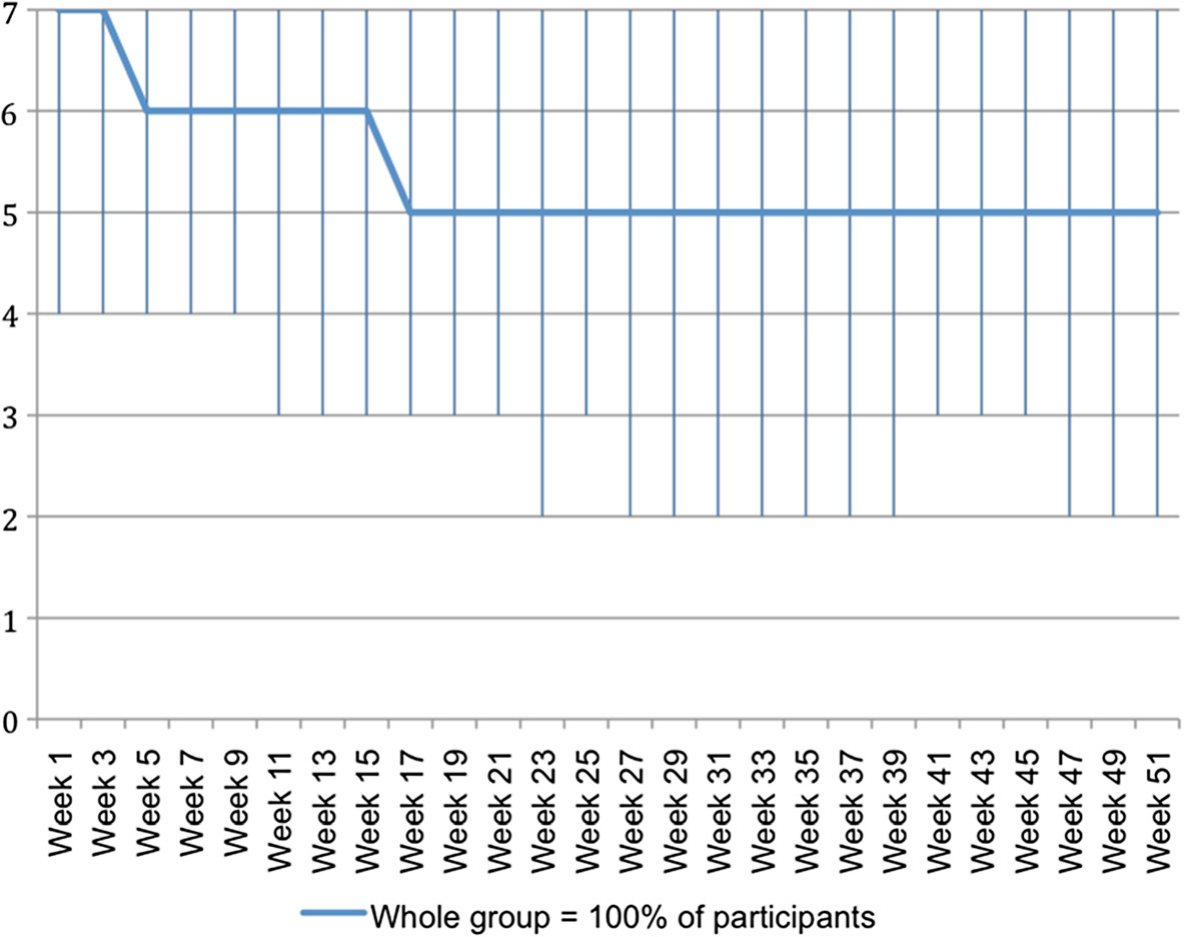
**Days per week (0 to 7) with problems due to back pain – pattern showing the clinical course (median) of the whole group using** *** fortnightly *** **SMS data.**

For the whole cohort, the clinical course pattern for pain problems is shown in Figure 3. These data show that, typically, people in the whole group started the study with 7 days of pain problems every week but by week 5 this had improved to 6 days per week, and further improved by week 18 to 5 days per week. After week 18, typically the group did not improve further. The variability in the participants is shown by the interquartile range.

**Figure 4:**
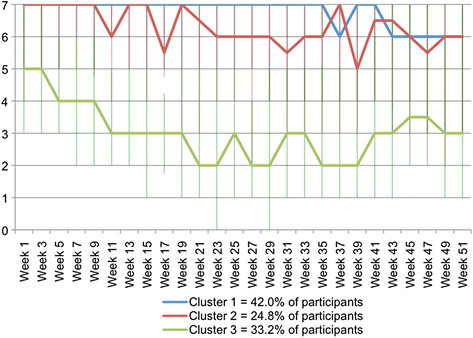
**Days per week (0 to 7) with problems due to back pain – clinical course patterns identified using Two-Step Cluster Analysis of** *** fortnightly *** **SMS data.**

The clinical course patterns for pain problems that were identified with cluster analysis of *fortnightly* data are shown in Figure 4. In contrast to the pattern for the whole group, these clusters show three different patterns. One cluster, that contained a third of the participants, typically had 5 days per week with pain problems at baseline and this improved by week 20 to be 2 to 3 days per week. The number of days remained at approximately this level for the rest of the observation period. A second cluster, that contained 2 out of 5 participants, typically had 7 days per week with pain problems at baseline and experienced no improvement until week 37. Thereafter, there was an improvement to 6 days per week. The third cluster, which contained one in four participants, typically had 7 days per week with pain problems at baseline and had an early fluctuating improvement to 6 days per week.

**Figure 5:**
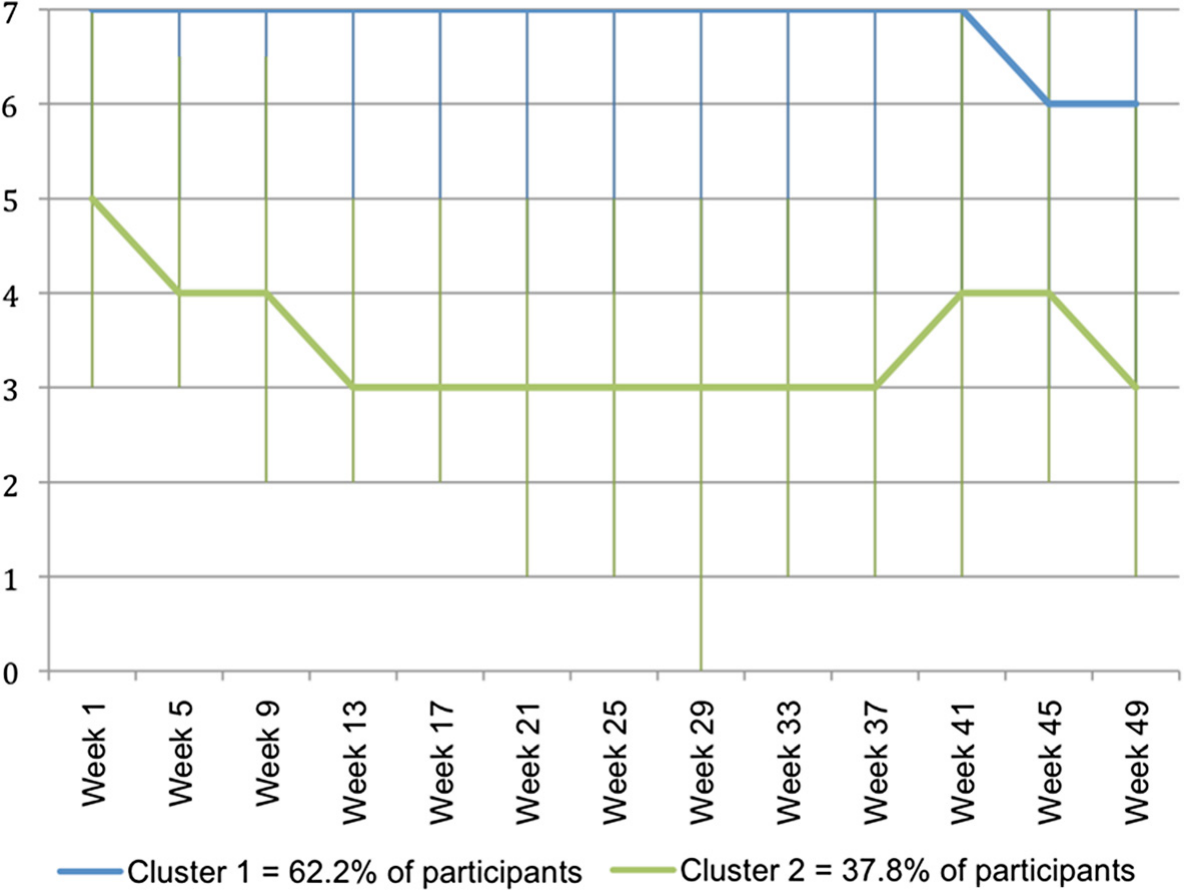
**Days per week (0 to 7) with problems due to back pain – clinical course patterns identified using Two-Step Cluster Analysis of** *** monthly *** **SMS data.**

Figure 5 shows the clinical course patterns for pain problems that were identified with cluster analysis of *monthly* data. Fewer data points resulted in similar but not identical clinical courses, as the two most similar clinical course patterns seen when using fortnightly data were joined into one clinical course pattern when using monthly data. This is because the reduced frequency of the data available was inadequate to identify differences between these two patterns.

### Clinical course of days with sick leave

**Figure 6:**
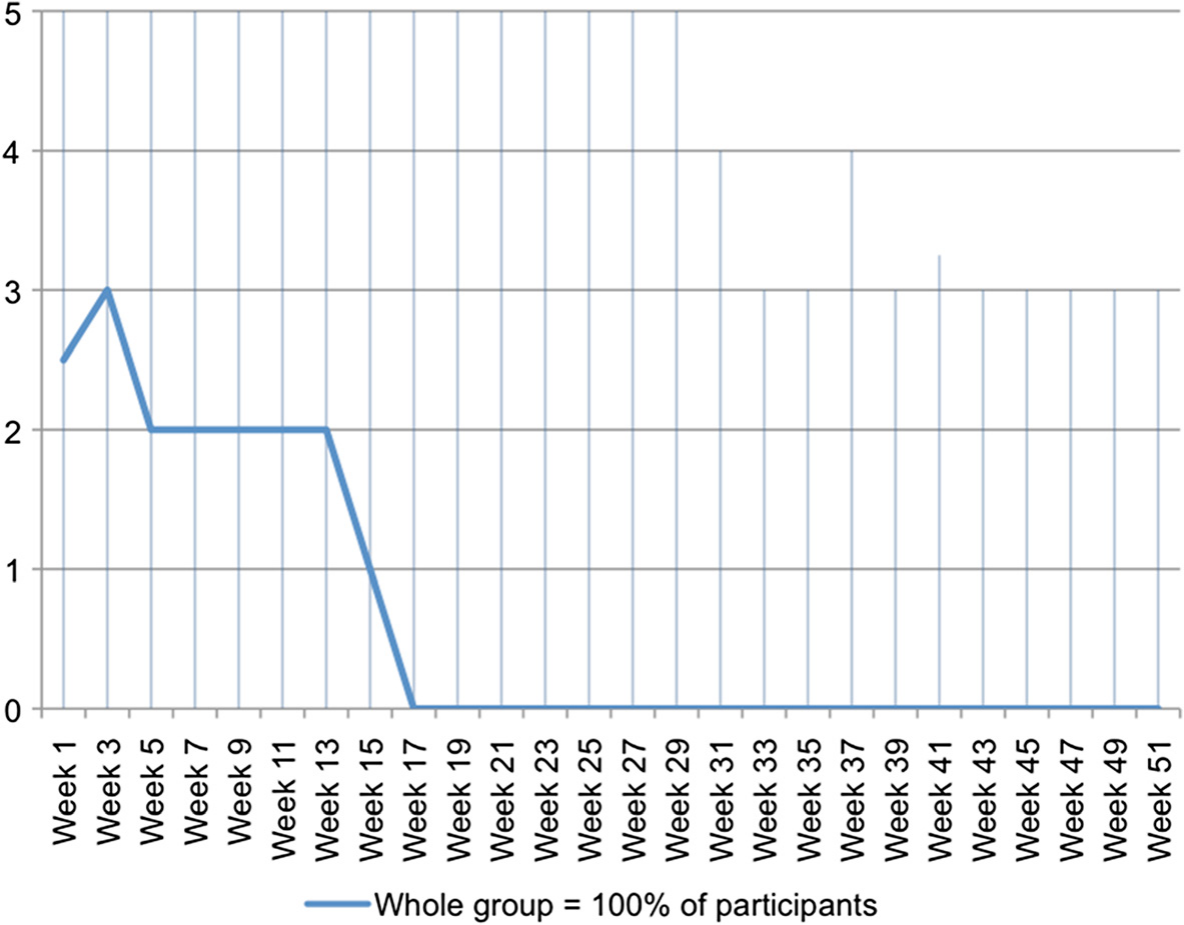
**Days per working week (0 to 5) of sick leave due to back pain – pattern showing the clinical course (median) of the whole group using** *** fortnightly *** **SMS data.**

For the whole cohort, the clinical course pattern for sick leave is shown in Figure 6. These data show that, typically, people in the whole group started the study with approximately 2.5 days of sick leave per week and by week 5, this improved to 2.0 working days of sick leave per week. By week 13, this rapidly improved and by week 17, people in the whole group typically had no days off work due to sick leave.

**Figure 7:**
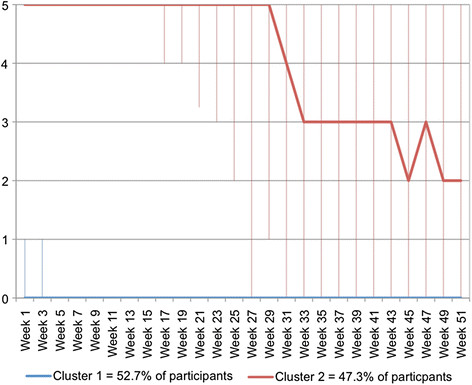
**Days per working week (0 to 5) of sick leave due to back pain –clinical course patterns identified using Two-Step Cluster Analysis of** *** fortnightly *** **SMS data.**

The clinical course patterns for sick leave that were identified with cluster analysis of *fortnightly* data are shown in Figure 7. In contrast to the pattern for the whole group, these clusters show two different patterns. In one cluster, that contained just over half of the participants, people typically took no sick leave during the year they were studied. In contrast, people in the other cluster typically had 5 days of sick leave per week from the start of the observation period right through to week 28. Thereafter, there was an improvement in their participation and by week 33 they typically had 3 days of sick leave per week. By week 2, this typically was 2 days of sick leave per week.

**Figure 8:**
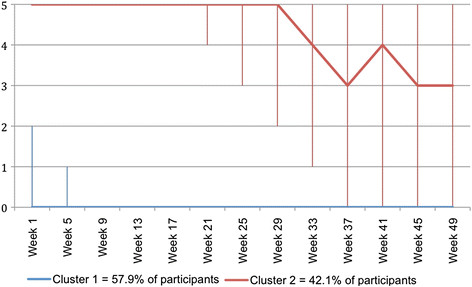
**Days per working week (0 to 5) of sick leave due to back pain – clinical course patterns identified using Two-Step Cluster Analysis of** *** monthly *** **SMS data.**

Figure 8 shows the clinical course patterns for sick leave that were identified with cluster analysis of *monthly* data. These patterns are very similar to those obtained with fortnightly data, though the median number of days of sick leave for the second group was 3 per week by week 49, whereas it was 3 per week by week 33 in the fortnightly data.

The collinearity of the *fortnightly* SMS pain data had a mean of 0.59 (SD0.16, range 0.23 to 0.89) and the *monthly* SMS pain data had a mean of 0.57 (SD0.15, range 0.23 to 0.83). The collinearity of the *fortnightly* SMS sick leave data had a mean of 0.58 (SD0.15, range 0.30 to 0.90) and the *monthly* SMS sick leave data had a mean of 0.57 (SD0.15, range 0.31 to 0.85).

## Discussion

This ‘proof of concept’ study shows that a probabilistic form of cluster analysis can be used for identifying clinical course patterns using high frequency longitudinal data such as SMS. It used one type of probabilistic cluster analysis (SPSS Two-Step) and a novel method for handling the raw data from frequently repeated measures (using all the SMS time points as cluster variables).

There are other statistical pattern-recognition approaches available for clustering SMS data, including latent class analysis [14], probabilistic data-mining [23], classical cluster analysis [16] and neural networks [24]. We chose to use Two-Step cluster analysis because it is a probabilistic-based method that is readily available in the base version of SPSS and has a shorter learning curve than some alternative approaches. However, there are few head-to-head comparisons of these approaches that can guide decisions on method selection [25,26] and none using SMS data.

Similarly, there are other statistical approaches available for distinguishing clinical course patterns in SMS data, including the clustering of regression coefficients of individual clinical courses that have been calculated using spline analysis [11]. However, the method used in the current study is simple, intuitive and does not require a high level of statistical sophistication.

We identified clinical course patterns using *fortnightly* SMS data and also using *monthly* SMS data. The patterns were similar but not identical. Further research should be performed to provide guidance on the statistical and practical implications of different frequencies of SMS data collection and for measurements in diverse health domains.

### Implications of clinical course patterns

The clinical course patterns identified in this study are intended only to demonstrate the utility of a particular statistical approach. As the cohort was formed from a convenience sample of two randomised controlled trials, these patterns are not intended to be generalisable to other cohorts.

Clinical course patterns may or may not provide useful information about clinically important subgroups. Clinical course is an outcome and that outcome is usually the product of many effects. For example, in the two trials that formed the cohort used in this study, all treatment groups did improve but there were no statistically significant differences between the outcomes achieved by each treatment group. That improvement is likely to be due to a composite of natural history, prognostic factors, non-specific treatment effects, and actual treatment effects that were equal between groups. On their own, these clinical course data do not provide any insight into the relative contribution of those components.

Therefore, clinical course patterns are a form of exploratory hypothesis-setting that require further investigation to determine if they contain any clinically meaningful information. For example, one approach is to use regression models with cluster membership as the dependent variable to identify baseline characteristics that distinguish cluster membership. In the context of a controlled trial, that regression analysis should include treatment group. Put simply, while SMS data can provide a more detailed estimate of clinical course patterns than can be provided by an outcome measured at a single point in time, such patterns need to be rigorously investigated for meaning and interpreted with caution. If such patterns do not separate people at baseline in clinically useful ways or on treatment exposure, then maybe they don’t have any clinical relevance.

### Methodological considerations

Different statistical approaches have different requirements (missing data, overfitting, normality and other aspects of data distribution) and these may influence the clinical course patterns obtained with such cluster methods. Though beyond the scope of the current study, head-to-head comparisons of multiple datasets would be useful to determine the relative merits of different statistical approaches.

The method used in this study required that the 14.2% missing SMS data be imputed but thereby allowed all individuals to be allocated to a cluster. Theoretically, the spline analysis approach used by Axen et al. [11] should cope better with missing data, although due to a number of methodological considerations including missing data, 41.2% of individuals were excluded from the final clusters in that study. Of course, imputation could be used prior to spline analysis to increase the proportion of participants clustered. An alternative approach would be to use statistical clustering techniques that tolerate missing data, for example, latent class analysis [14] and probabilistic data-mining [23].

A further consideration is that independence of included variables is an assumption of many cluster techniques. This is because highly correlated (collinear) variables can distort the cluster model if the other variables are not highly correlated. The SMS data in our study had a global correlation of approximately 0.58 across all the time points (full range approximately 0.25 to 0.86). It is not known if this is an important consideration that should affect the choice of how to manage SMS data. It may be that if most of the variables share a relatively narrow range of global correlation, this does not significantly distort the cluster model. Hypothetically, collinearity of SMS data would be reduced by using regression coefficients calculated using spline analysis instead of the data in its original format but this needs to be quantified, preferably in multiple datasets.

The cluster analysis, dataset and method for handling SMS data used in the current study collectively resulted in fewer clinical course patterns than described by Axen et al. [11]. One reason could be that different cluster analysis techniques may have varying sensitivity, and therefore describe different numbers of patterns, even when handling SMS data in exactly the same way. Alternatively, it may be that spine analysis is more sensitive than clustering all the original data, though this is unlikely. Lastly, it may be due to our sample being from secondary care, rather than the primary care setting sample by Axen et al. Conceptually, the optimal number of clinical course patterns would depend not only on the inherent characteristics of the available longitudinal data but also on how clinically useful are the distinctions between people in each cluster.

Based on our observation and opinion, Table 2 contains a summary of the advantages and disadvantages offered by clustering SMS data using all time points compared with clustering SMS regression coefficients. These opinions may not all be correct and should be tested in subsequent studies that use multiple samples, of differing variance, with varying frequency of SMS sampling, and differing types of cluster methods. An additional consideration is whether in SMS data, due to the frequency of sampling, a ‘last-value-forward’ is a more accurate way to handle missing data than multiple imputation.

**Table 2:** The advantages and disadvantages provided by clustering SMS data using either of two different formats

	**SMS data in original format (all SMS time points used for clustering)**	**SMS data transformed into regression coefficients by spline analysis**
Simple and intuitive	✓	
Copes with data that do not show a time trend	✓	
Copes with data from clinical course patterns that are fluctuating	✓	
Copes with clinical course data that are all zero values or the same value at all time points	✓	
Preserves all the original information in the data	✓	
With imputation of missing data, all cases can be included, regardless of clinical course patterns	✓	
Copes better with missing data		✓
A data reduction technique (reduces the likelihood of overfitting the data)		✓
Reduces the collinearity (autocorrelation) of the data		✓
Requires pre-hoc assumptions about which spline characteristics are clinically important. This may improve interpretability but also may introduce bias, and require the exclusion of cases that do not meet those assumptions		✓

In this study, we transformed the SMS data to approximate normality before the cluster analysis occurred. There are contradictory reports published about the assumptions of data distribution inherent in particular clustering techniques and their robustness to non-normality. Lacking are published studies which actually demonstrate that robustness and one reason for this may be that, because cluster analysis is exploratory, there is a lack of an appropriate reference standard to make that judgement. One approach would be to compare the cluster membership of people classified using different cluster techniques and determine whether there is greater classification similarity when the data approximates normality. We have performed such (unpublished) analyses and those results suggest that in our data, cluster techniques such as 2-Step Cluster Analysis and probabilistic data-mining reach more similar conclusions about the cluster membership of individuals when the data approximates normality. The exception is when the class structure is very simple. Of course, a disadvantage of transforming data is the need to re-interpret the results using the original form of data, as the transformed data is usually not readily interpretable from a clinical perspective.

Some of the methodological and interpretive issues considered in this study are likely to also apply to the analysis of repeated measures obtained via methods other than SMS and also to measures obtained at lower frequencies. For example, two studies have used latent class analysis to examine clinical course patterns in pain data, obtained via weekly pain diaries [14] or via monthly questionnaires[15]. Using a different approach, Chen et al. [12] described clinical course patterns in pain intensity scores acquired in interviews at baseline, 4, 16 and 52 weeks. In that study the researchers, using simple linear regression, first calculated the slope of recovery for each individual and stratified those slopes into three patterns. They then used k-means cluster analysis to identify clinical course patterns within each group.

We believe there is a need to undertake further research to clarify which statistical approaches might be optimal for identifying clinical course patterns in high frequency repeated measures data, and in what circumstances. An advantage of this knowledge would be a standardisation of methods that would better allow comparisons of the results obtained in different studies. As clinical course patterns are a form of hypothesis-setting, replication studies using the same methodology are an important aspect of validation.

### Strengths and weaknesses of study

The strengths of this ‘proof of concept’ study are that real SMS data were used to explore a new method for identifying clinical course patterns, more than one health domain was modeled (pain and sick leave), and the impact of different frequencies of SMS measurements was investigated (fortnightly and monthly). The weaknesses of this study are that only a single data set was used, there are other statistical approaches that could have been used, and there remain many unanswered questions about which clustering approach is most appropriate in particular circumstances.

## Conclusions

This study showed that clinical course patterns can be identified by cluster analysis that includes all SMS time points in longitudinal data to identify cluster patterns. This method is simple, intuitive and does not require a high level of statistical skill. However, there are alternative ways of managing SMS data and many different types of cluster analysis techniques. More research is needed to identify which technique is best to use under what circumstance

## Abbreviations

LBP, Low Back Pain; SMS, Short Message Service (text messaging).

## Competing interests

The manuscript submitted does not contain information about medical devices or drugs. No benefits in any form have been, or will be, received from a commercial party related directly or indirectly to the subject of this manuscript.

## Authors’ contributions

PK and AK were involved in the design of the study, interpretation of data, revision of the manuscript, and gave final approval of the manuscript. PK performed the data analysis and wrote the initial draft of the manuscript. All authors read and approved the final manuscript.
